# Is the ratio superior to the number of metastatic lymph nodes in addressing the response in patients with papillary thyroid cancer?

**DOI:** 10.1097/MD.0000000000009664

**Published:** 2018-01-19

**Authors:** Wen Gao, Teng Zhao, Jun Liang, Yansong Lin

**Affiliations:** a Department of Nuclear Medicine, Peking Union Medical College Hospital, Beijing; b Department of Oncology, the Affiliated Hospital of Qingdao University, Qingdao; c Department of Otorhinolaryngology Head and Neck Surgery, Beijing Shijitan Hospital, Capital Medical University; d Department of Oncology, Peking University Cancer Hospital and Institute, Beijing, China.

**Keywords:** clinical response, lymph node metastatic ratio, number of metastatic lymph nodes, papillary thyroid cancer

## Abstract

**Context:**

The number of metastatic lymph nodes (LNs) and the ratio of metastatic LN (LR) have been reported as predictors of recurrence in papillary thyroid carcinoma (PTC), while the role of LR or the number of metastatic LNs on the clinical response remains unclear.

**Objective:**

We aimed to compare the prognostic value of LR and the number of metastatic LNs on clinical response in PTC.

**Design/Setting/Patients:**

A total of 384 PTC patients with LN metastases were enrolled in this study, all of whom underwent total or near total thyroidectomy and subsequent radioiodine ablation.

**Main Outcome Measures:**

After a mean follow-up of 25.7 months, response to initial therapy was classified as excellent response (ER), indeterminate, biochemical incomplete or structural incomplete response. The scatter diagram and receiver operating characteristic (ROC) curve were respectively employed to identify and compare the clinical value of the number of metastatic LNs and LR for predicting ER in different number of dissected LNs (DLNs). Multivariate analyses were further performed to explore the indicator for ER.

**Results:**

ER tended to be more concentrate in patients with lower LR and lower number of metastatic LNs in scatter diagram. Although in patients with ≤10 DLNs, LR presented higher area under the ROC curve than the number of metastatic LNs in predicting ER (LR: 0.687, LNs, 0.556, *P* = .02), whereas it turns opposite in those with >10 DLNs. In the multivariate analysis, LR (odds ratio [OR] = 1.037, *P* = .001) rather than the number metastatic LNs (OR = 0.752, *P* = .09) was an independent indicator for ER in addition to preablative-stimulated thyroglobulin (ps-Tg; OR = 1.056, P = .01) among patients with ≤10 DLNs. Although in patients with >10 DLNs, the number of metastatic LNs (OR = 1.062, *P* = .04) turned to be independent factor for ER, apart from ps-Tg (OR = 1.071, *P* = .00) and sex (OR = 0.570, *P* = .02).

**Conclusions:**

LR appears to be a better negative predictor for ER than the number of metastatic LNs in PTC patients with ≤10 DLNs, whereas the number of metastatic LNs is superior to LR in those with >10 DLNs.

## 1 Introduction

Lymph node (LN) metastasis occurs in up to 90% of patients with papillary thyroid carcinoma (PTC).^[1]^ Several authors have described the deleterious effect of LN metastasis on survival and recurrence.^[2,3]^ Location, extranodal extension, size, especially the number of metastatic LNs and LN metastatic ratio (LR) are relevant manifestations of LN metastases.^[4–7]^ The number of metastatic LNs is 1 of the common indicators to address the involvement of LN metastases, the higher the number of involved LNs, the higher incidence of the recurrence.^[6]^ Our recent research indicated that 10 or more dissected LNs (DLNs) might reflect adequate intraoperative evaluation and carry incremental value in achieving better response.^[8]^ Although for LR, which refers to the involved ratio of metastatic LNs, it may afford more information regarding the intraoperative evaluation range. One Korean study showed that LR is a significant risk factor for predicting poor prognosis.^[9]^ In 2015 American Thyroid Association (ATA), a new therapeutic response system is proposed to specify the recurrence and mortality in patients after both total/near total thyroidectomy and radioiodine (RAI) therapy, which appears to be a more objective ongoing assessment in predicting the clinical prognosis.^[10]^ It remains uncertain whether LR holds more value for both therapeutic response and clinical prognosis in PTC patients when comparing with the number of metastatic LNs. This study aimed to elucidate the predictive value of LN metastasis for clinical outcome in terms of LR and the number of metastatic LNs.

## 2 Materials and methods

### 2.1 Patients

We retrospectively studied a total of 985 patients diagnosed with PTC, patients underwent postoperative RAI ablation between October 2012 and December 2014 in Chinese Academy of Medical Science, all of whom underwent total or near total thyroidectomy with LN dissection. Among them 601 patients were excluded because of any of the following reasons: with initial distant metastases; with no evidence of LN metastasis on histology; (3) inadequate follow-up information for at least 6 months/incomplete follow-up records after 1st RAI therapy. Furthermore, written consent was obtained from the patients in the present study.

### 2.2 Initial therapy

Total or near total thyroidectomy was usually performed in patient who meets either of the follows: primary tumor >1 cm; presence of multiple or bilateral lesions; extrathyroid extension; suspicious metastatic LNs during the preoperative evaluation or surgery. RAI ablation was employed following levothyroxine (LT4) withdrawal or with no LT4 therapy after surgery, accompanied with a low-iodine diet for at least 2 weeks as per our normal clinical protocol. Dose of RAI ranged from 1.11 GBq (30 mCi) to 5.55 GBq (150 mCi) was delivered according to tumor stage and likelihood of recurrence as per risk stratification. Routine medical examinations before RAI ablation included serum thyrotropin (TSH), preablative-stimulated thyroglobulin (ps-Tg), anti-Tg antibody (TgAb), chest computed tomography (CT), neck ultrasound (US), and diagnostic RAI whole-body scan (DxWBS), if necessary. Posttherapy RAI whole-body scan was performed 5 to 8 days after therapeutic RAI administration.

### 2.3 Follow-up protocol

After initial therapy, patients took LT4 for TSH suppression after 3 days. Patients also received regular follow-up with examinations, such as neck US and DxWBS as well as serum-stimulated thyroglobulin level with TgAb performed 6 to 12 months after RAI therapy. Additional diagnostic imaging studies, such as chest CT and whole body fluoro-deoxyglucose-PET were performed if needed. Considering the status of LN evaluation, patients were divided into 2 cohorts according to the number of DLNs.

### 2.4 Evaluation of response to initial therapy

Patients response after initial RAI ablation were classified into 4 types: excellent (ER), indeterminate (IDR), biochemical incomplete (BIR), and structural incomplete (SIR) according to the reclassification system newly proposed by ATA.^[9]^ Patients who achieved ER should accord with the criteria below: either suppressed Tg <0.2 ng/mL or stimulated Tg <1 ng/mL, US without evidence of disease, and negative cross-sectional and/or nuclear medicine imaging (if performed). The criteria for IDR is defined that if patients had any of the following: nonspecific findings on imaging studies with faint uptake in thyroid bed on RAI scanning, suppressed Tg ≥0.2 ng/mL but <1 ng/mL, stimulated Tg ≥1 but <10 ng/mL, or TgAb stable or declining in the absence of structural or functional disease. BIR was defined with either of the following criteria: negative imaging and suppressed Tg >1 ng/mL, or stimulated Tg >10 ng/mL, or rising TgAb values. SIR represented the patients who had structural or functional evidence of disease with any Tg level and/or TgAb.

### 2.5 Statistical analysis

Data were presented as median rang or mean ± standard deviation. Scatter diagram was used to identifying the patients who showed ER under different LR and number of metastatic LNs in different context of DLNs (≤10 or >10). Moreover, receiver operating characteristic (ROC) curve was applied to determine the area under the curve (AUC) to evaluate the value of LR and the number of metastatic LNs in predicting ER in different context of DLNs. Comparison of ROC curve was performed using the method of Hanley and McNeil for the same set of patients.^[10]^ Multivariate logistic regression analyses were further analyzed among patients with ≤10 or >10 DLNs, to find independent prognostic factors for ER, including sex, age, T stage, N status, LR, the number of metastatic LNs, ps-Tg, and dose of RAI. All tests were 2 sided, and a *P* value <.05 was considered statistically significant. Statistical analyses were performed using the SPSS version 22.0 and MedCalc version 14.8.1.

## 3 Results

### 3.1 Clinicopathologic characteristics of patients

Characteristics of the patient are summarized in Table 1. A total of 384 patients were enrolled in this study, including 132 men (34.4%) and 252 women (65.6%). Of all the patients, 272 patients were dissected at least 10 LNs or more, whereas the remaining 112 patients underwent <10 LN dissection. The median follow-up period was 25.7 months.

**Table 1:**
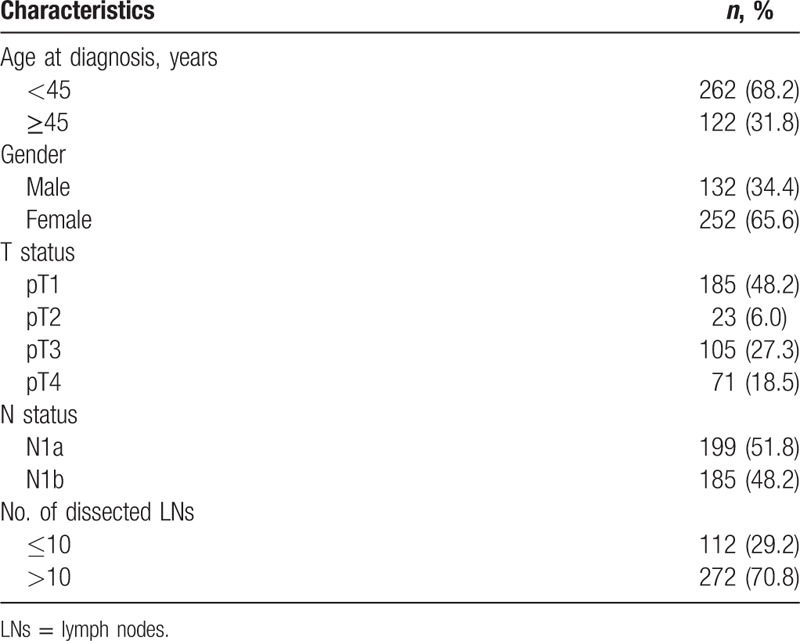
Clinicopathologic characteristics in patients with papillary thyroid cancer, *n* = 384.

### 3.2 Comparison between LR and the number of metastatic LNs when the number of DLNs ≤10

**Figure 1:**
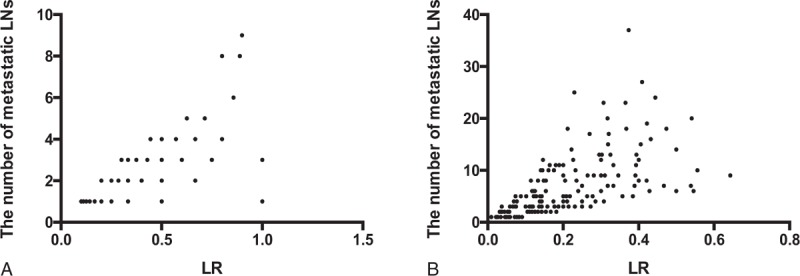
The distribution of patients with excellent response under different lymph node metastatic ratio and number of metastatic lymph nodes when the number of dissected lymph node below 10 (A) and above 10 (B). LNs = lymph nodes, LR = lymph node metastatic ratio.

**Figure 2:**
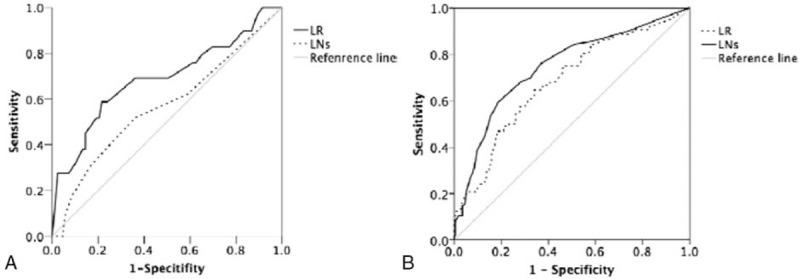
The ROC curves of the number of metastatic LNs and LR in predicting excellent response when the number of dissected lymph node below 10 (A) and above 10 (B). LNs = lymph nodes, LR = lymph node metastatic ratio.

With the decreasing of LR and number of metastatic LNs, the number of patients with ER simultaneous mounting in Fig. 1A. As shown in Fig. 2A and Table 2, LR, with an AUC of 0.687 and a cut-off value of 0.59, rather than the number of metastatic LNs, was demonstrated as a prognostic marker in predicting ER (LR: 0.687; LNs:0.566). Multivariate logistic regression also showed that LR (odds ratio [OR] = 1.036, 95% CI: 1.014–1.058, *P* = .001) was an independent negative indicator for ER in addition to Ps-Tg (OR = 1.057, 95% CI: 1.104–1.102, *P* = .009; Table 3).

**Table 2:**
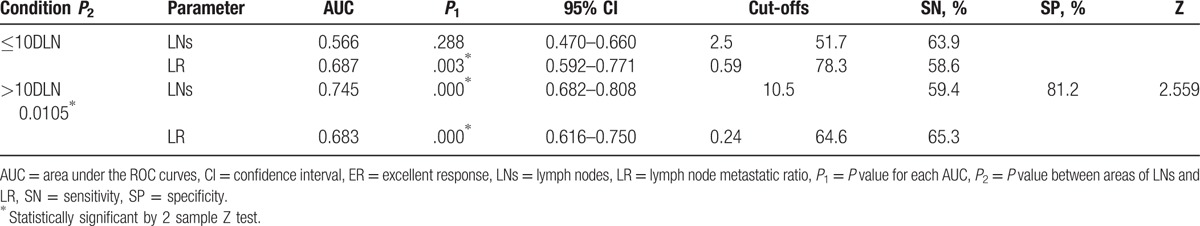
Results of ROC analyses for the number of metastatic LNs and lymph node metastatic rate in identifying ER and non-ER in different dissected yield.

**Table 3:**
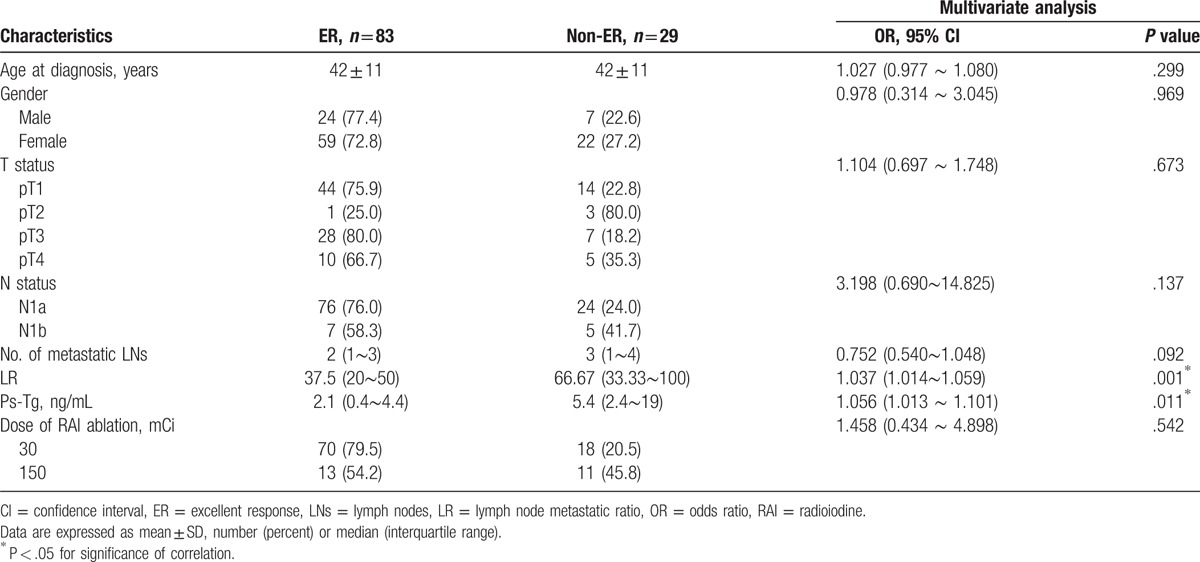
Multivariate analyses of prognostic indicators for ER when the number of dissected lymph node below 10.

### 3.3 Comparison between LR and the number of metastatic LNs when the number of DLNs >10

For this part of patients, who presented ER continued to rise over the course of LR and the number of LNs increasing (Fig. 1B). Contrary to the results in patients with ≤10 DLNs, the AUC of the number of metastatic LNs was significantly higher than LR in predicting ER in those with >10 DLNs (LNs: 0.745; LR: 0.683; *P* = .01; see Fig. 2B; Table 2). The potential-associated factors for ER were shown in Table 3. In multivariate analysis, the number of metastatic LNs (OR = 1.086, 95% CI: 1.030–1.145, *P* = .002), ps-Tg (OR = 1.067, 95% CI: 1.034–1.101, *P* = .00), and sex (OR = 0.570, 95% CI: 0.351–0.925, *P* = .02) were demonstrated to be the independent predictive factors for ER (Table 4).

**Table 4:**
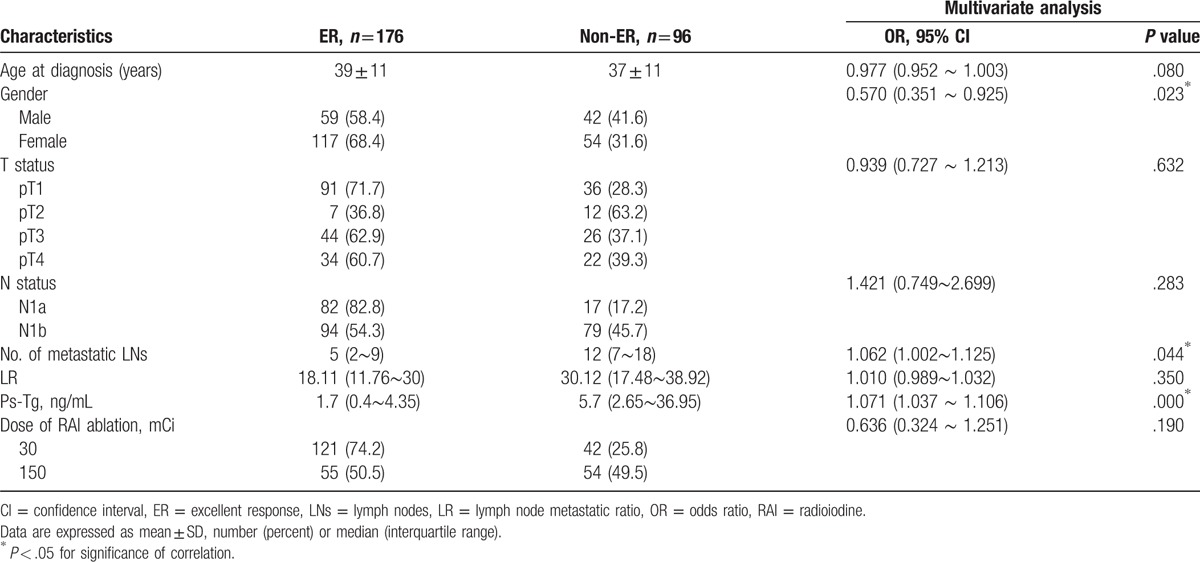
Multivariate analyses of prognostic indicators for ER when the number of dissected lymph node above 10.

## 4 Discussion

Though carrying a good 10-year survival rate at 93%, an increased risk of recurrence and compromised survival rate can be observed in PTC patients with nodal involvement.^[6,13]^ Surgical dissection and subsequent selective RAI ablation are therefore recommended as the 1st line protocol, with an aim to reduce regional recurrence in patients with intermediate or high risk of recurrence.^[14,15]^ Still, even after initial treatment, cervical recurrence could occur in up to 30% of patients.^[14]^ Invasive extent of LN metastases including the number of metastatic LNs and LR has been shown to be 1 of poor factor for recurrence.^[16]^ Study from Leboulleux et al^[6]^ revealed that the 10-year locoregional recurrence rate would rise from 3%, 7 to 21% among patients with the number of metastatic LNs <5, 6 to 10 and >10, respectively. Sugitani I et al^[17]^ reported that the recurrence rate of patients with any metastatic LN >3 cm would be 27%. Based upon these studies, the 2015 ATA guideline 1st takes the number of metastatic LNs and size of LN metastases into account when stratifying the recurrence risk of patients,^[10]^ while without mentioning the context of DLNs. LR has recently emerged as an alternative prognostic indicator, which compromises both the number of metastatic LNs and DLNs. A recent report indicated that the 5-year locoregional recurrence-free survival were 97.1% and 78.8% in patients with LR below or above 0.22, respectively.^[9]^ The range of the neck dissection has been the concern of the surgeons, our recent research indicated 10 or more DLNs seems to carry incremental value in achieving better response.^[8]^ About 10, DLNs, therefore, could be regarded as a reference for proper number for intraoperational LN evaluation. To our knowledge, no reports have valued LR and the number of metastatic LNs in predicting clinical outcome, especially in terms of the new clinical response system raised by 2015 ATA guidelines. In this study, for the 1st time, we tried to compare the prognostic impact between LR and the number of metastatic LNs on clinical response under different LN dissection yield.

Our study finds that ER tends to occur in lower LR or less number metastatic LNs in regardless the number of DLNs. Moreover, it is worthy to mention that LR, rather than the number of metastatic LNs, is a prognostic indicator for ER in patients with less than 10 DLNs. Conversely, in patients with the number of DLNs more than 10, the number of involved LNs manifested leading role in predicting ER. Multivariate analyses also suggested that the number of involved LNs, rather than LR, became an independent factor for ER. What may contribute to the difference between the 2 circumstances? One possible explanation seems that more DLNs may provide a kind of ‘more adequate LN evaluation,’ so that the number of metastatic LNs could play an informative and objective role in presenting the actual extent of LNs metastasis. In contrast, in patients with fewer DLNs, the number of metastatic LNs might be less objective because of relatively more uncertainty remained in those unresected LNs, in this case, LR appears to be a more comprehensive indicator taking both the number and the extent of LN metastasis into consideration. Thus, this system in combination of LR and the number of metastatic LNs might be helpful for evaluating both LN management and optimal postoperative prognostic system for PTC.

Like the results indicated in our previous work,^[18]^ ps-Tg is also an independent factor for ER, regardless of the LN dissection yield. Furthermore, age is also an independent factor when more LNs are evaluated, which implies that the older patients are easy to exhibiting non-ER. This is inconsistent with Chéreau et al,^[19]^ who have demonstrated that the patients with advanced age have a negative effect on prognosis. In addition, we observed that the dose of RAI ablation was not significantly related to clinical response in our population, which might provide more evidence to reinforce that 30 mCi is effective enough for remnant ablation in intermediate risk patients.^[20]^

Although we enrolled and analyzed 384 patients, this study still had several limitations related to sample size as well as its retrospective nature. In addition, number of metastatic LNs detected is dependent on the preoperative assessment, surgical skills, and surgery management strategies.

## 5 Conclusion

In conclusion, LR may serve as a better marker for an alternative staging system than the number of LNs in patients with ≤10 DLNs, whereas the number of metastatic LNs models demonstrated superior prognostic value to LR in those with >10 DLNs. Our findings suggest a comprehensive LN evaluation combining both LR and number of metastatic LNs, which might be a new optimal prognostic system for PTC.
